# Characterization and risk estimate of cancer in patients with primary Sjögren syndrome

**DOI:** 10.1186/s13045-017-0464-5

**Published:** 2017-04-17

**Authors:** Pilar Brito-Zerón, Belchin Kostov, Guadalupe Fraile, Daniel Caravia-Durán, Brenda Maure, Francisco-Javier Rascón, Mónica Zamora, Arnau Casanovas, Miguel Lopez-Dupla, Mar Ripoll, Blanca Pinilla, Eva Fonseca, Miriam Akasbi, Gloria de la Red, Miguel-Angel Duarte-Millán, Patricia Fanlo, Pablo Guisado-Vasco, Roberto Pérez-Alvarez, Antonio J. Chamorro, César Morcillo, Iratxe Jiménez-Heredia, Isabel Sánchez-Berná, Armando López-Guillermo, Manuel Ramos-Casals, on behalf of the SS Study Group GEAS-SEMI, M. Ramos-Casals, P. Brito-Zerón, S. Retamozo, A. Bové, H. Gheitasi, I. Sánchez-Berná, J. Gratacós, G. Fraile, J. Nava-Mateos, B. Díaz-López, D. Caravia-Duran, A. García-Pérez, A. Casanovas, M. L. Morera-Morales, T. E. Junco-Russeau, F. J. Rascón, L. Pallarés, R. Pérez-Alvarez, M. Perez-de-Lis, M. Ripoll, C. Moreno-delaSanta-García, B. Pinilla, C. López-GónzalezCobos, M. Akasbi, I. García-Sanchez, B. Maure, E. Fonseca, M. A. Duarte-Millán, J. Canora, G de la Red, N. Msabri, A. J. Chamorro, S. Rodríguez-Rodríguez, I. Jiménez-Heredia, M. A. López-Dupla, J. A. Porras-Ledantes, B. Villar-Navas, J. Ramos-Rodriguez, P. Brito-Zerón, C. Morcillo, M. Zamora, I. Sánchez-Berná, P. Fanlo, P. Guisado-Vasco, B. Kostov, A. Sisó-Almirall

**Affiliations:** 1 Autoimmune Diseases Unit, Department of Internal Medicine, Hospital CIMA-Sanitas, Barcelona, Spain; 2 Laboratory of Autoimmune Diseases Josep Font, IDIBAPS, Department of Autoimmune Diseases, ICMiD, Hospital Clínic, Barcelona, Spain; 3 Transversal group for research in primary care, IDIBAPS, Consorci d’Atenció Primària de Salut Barcelona Esquerre (CAPSBE), Barcelona, Spain; 4 Department of Internal Medicine, Hospital Ramón y Cajal, Madrid, Spain; 5 Department of Internal Medicine, Hospital Universitario Central de Asturias, Oviedo, Spain; 6 Department of Internal Medicine, Complejo Hospitalario Universitario, Vigo, Spain; 7 Department of Internal Medicine, Hospital Son Espases, Palma de Mallorca, Spain; 8 Department of Internal Medicine, Hospital Virgen de las Nieves, Granada, Spain; 9 Department of Internal Medicine, Hospital Parc Taulí, Sabadell, Spain; 10 Department of Internal Medicine, Hospital Joan XXIII, Tarragona, Spain; 11 Department of Internal Medicine, Hospital Infanta Sofía, Madrid, Spain; 12 Department of Internal Medicine, Hospital Gregorio Marañón, Madrid, Spain; 13 Department of Internal Medicine, Hospital de Cabueñes, Gijón, Spain; 14 Department of Internal Medicine, Hospital Infanta Leonor, Madrid, Spain; 15 Department of Internal Medicine, Hospital Esperit Sant, Santa Coloma de Gramenet, Spain; 16 Department of Internal Medicine, Hospital de Fuenlabrada, Fuenlabrada, Spain; 17 Department of Internal Medicine, Hospital Virgen del Camino, Pamplona, Spain; 18 Department of Internal Medicine, Complejo Hospitalario Ruber Juan Bravo, Madrid, Spain; 19 Department of Internal Medicine, Hospital Alvaro Cunqueiro, Vigo, Spain; 20 Department of Internal Medicine, Hospital de Salamanca, Salamanca, Spain; 21 Department of Internal Medicine, Hospital de Sagunto, Valencia, Spain; 22 Department of Hematology, ICMHO, Hospital Clinic, Barcelona, Spain; 23 Department of Medicine, University of Barcelona, Barcelona, Spain

**Keywords:** Sjögren syndrome, Cancer, Lymphoma

## Abstract

**Background:**

The purpose of this study is to characterize the risk of cancer in a large cohort of patients with primary Sjögren syndrome (SjS).

**Methods:**

We had analyzed the development of cancer in 1300 consecutive patients fulfilling the 2002 SjS classification criteria. The baseline clinical and immunological characteristics and systemic activity (ESSDAI scores) were assessed at diagnosis as predictors of cancer using Cox proportional hazards regression analysis adjusted for age at diagnosis and gender. The sex-and age-specific standardized incidence ratios (SIR) of cancer were estimated from 2012 Spanish mortality data.

**Results:**

After a mean follow-up of 91 months, 127 (9.8%) patients developed 133 cancers. The most frequent type of cancer was B-cell lymphoma (including 27 MALT and 19 non-MALT B-cell lymphomas). Systemic activity at diagnosis of primary SjS correlated with the risk of hematological neoplasia and cryoglobulins with a high risk of either B-cell or non-B-cell lymphoma subtypes. Patients with cytopenias had a high risk of non-MALT B-cell and non-B-cell cancer, while those with low C3 levels had a high risk of MALT lymphomas and those with monoclonal gammopathy and low C4 levels had a high risk of non-MALT lymphomas. The estimated SIR for solid cancer was 1.13 and 11.02 for hematological cancer. SIRs for specific cancers were 36.17 for multiple myeloma and immunoproliferative diseases, 19.41 for Hodgkin lymphoma, 6.04 for other non-Hodgkin lymphomas, 5.17 for thyroid cancer, 4.81 for cancers of the lip and oral cavity, and 2.53 for stomach cancer.

**Conclusions:**

One third of cancers developed by patients with primary SjS are B-cell lymphomas. The prognostic factors identified at SjS diagnosis differed according to the subtype of B-cell lymphoma developed. Primary SjS is also associated with the development of some non-hematological cancers (thyroid, oral cavity, and stomach).

**Electronic supplementary material:**

The online version of this article (doi:10.1186/s13045-017-0464-5) contains supplementary material, which is available to authorized users.

## Background

Sjögren syndrome (SjS) is a systemic autoimmune disease that principally affects women between the fourth and sixth decades of life who present with sicca symptomatology caused by dryness of the main mucosal surfaces [1]. The clinical spectrum of SjS extends from dryness to systemic involvement. Since 1978 [2], SjS has been closely associated with an enhanced risk of lymphoma, one of the most severe complications a patient may develop. Primary SjS patients have a 10–44-fold greater risk of lymphoma than healthy individuals, higher than that reported for systemic lupus erythematosus (sevenfold) and rheumatoid arthritis (fourfold) [3]. The close link between lymphoma and SjS is clearly exemplified by the very specific type of lymphoma arising in SjS patients, mainly low-grade B-cell lymphomas (predominantly marginal zone histological type) with primary extranodal involvement of the major salivary glands (overwhelmingly parotid) [4, 5]. The lymphomagenesis hypothesis in primary SjS suggests a key role for the continued stimulation of B cells in the exocrine glands and organs containing mucosa-associated lymphoid tissue (MALT); in some patients, this autoimmune process is further altered by a combination of pro-oncogenic factors that promote abnormal increases in B-cell survival, thus putting the patient at high risk of a malignant B-cell transformation [6].

Studies that have analyzed the association between primary SjS and cancer have overwhelmingly been centered on the characterization and risk estimation of lymphoma [7]. Little data is available on the association between primary SjS and non-B-cell lymphomas [8–10] or solid cancer [11]. The aims of this study were to analyze the development of solid and hematological cancer, identify related baseline SjS- prognostic factors (including systemic activity), and estimate the corresponding standardized incidence ratios (SIR) per type of cancer in a large, well-characterized cohort of Spanish patients with primary SjS.

## Methods

### Patients

The GEAS-SS Study Group was formed in 2005 with the aim of collecting a large series of Spanish patients with primary SjS. Both incident and prevalent cases were included; for incident cases, the diagnosis of primary SjS was confirmed after January 2005. By January 2016, the database included 1300 consecutive patients (513 prevalent cases) who fulfilled the 2002 classification criteria for primary SjS [12]. Exclusion criteria were chronic HCV/HIV infection and associated systemic autoimmune diseases. Diagnostic tests for SjS (ocular tests, parotid scintigraphy, and salivary gland biopsy) were performed according to the European Community Study Group recommendations [12]. Clinical and laboratory data were collected and computerized according to a standard previously reported protocol [13].

### Definition of variables

The date of disease diagnosis was defined as the date when the attending physician confirmed fulfillment of the 2002 criteria [12]. Systemic involvement was defined according to the ESSDAI [14]. Disease activity states (DAS) were categorized according to the global ESSDAI score as low activity (ESSDAI <5), moderate activity (5 ≤ESSDAI≤ 13), and high-activity (ESSDAI ≥14) [15].

Cancers were determined through medical chart review of the hospital discharge summaries by the corresponding oncology/hematology departments. We included only cancers occurring after the confirmed diagnosis of primary SjS by the attending physician. Solid cancers were classified using the nomenclature included in the GLOBOCAN Project (http://globocan.iarc.fr/) and hematological cancers using the 2016 WHO classification [16, 17].

### Statistical analysis

Descriptive data are presented as means and standard deviation (SD) for continuous variables and numbers and percentages (%) for categorical variables. Time-to-event analyzes for cancer are presented as Kaplan-Meier curves. The baseline clinical and immunological characteristics and systemic activity (ESSDAI scores) were assessed at diagnosis as predictors of cancer using univariate Cox proportional-hazards regression analysis adjusted for age at diagnosis and gender. The level of systemic activity was recoded as no activity vs. any type of activity (low/moderate/high) in the analysis. Multivariate Cox proportional-hazards regression analysis allowed adjustment for age at diagnosis and gender and variables that were statistically significant (*p* < 0.05) in the univariate analysis, in order to establish independent variables associated with hematological and solid cancers. The hazard ratios (HRs) and their 95% confidence intervals (CIs) obtained in the adjusted regression analysis were calculated. The person-years of follow-up were calculated from the date of diagnosis to the last visit, cancer, or death (whichever occurred earliest). The standardized incidence ratios (SIR) were computed as the ratio of observed to expected cancers. Expected cancers were determined by multiplying person-years by the corresponding sex- and age-specific incidence rates of cancer in the general Spanish population in 2012 (the most recent data available from the International Agency for Research on Cancer) provided by the GLOBOCAN project (http://globocan.iarc.fr/old/method/method.asp?country=724) and summing overall person-years. The sex- and age-specific incidence rates of cancer for Spain were estimated from national mortality data for 2012 by modeling, using a set of age-, sex-, and site-specific incidence:mortality ratios obtained by the aggregation of recorded data from the 12 Spanish cancer registries [18]. The incidence rates of several types of cancers could not be estimated for population belonging to the age category “0–14” by the fact that these types of cancers were not detected in this population. Thus, this category was excluded when SIR were computed. The 95% confidence intervals (CI) of the SIR were also calculated. All significance tests were two tailed, and values of *p* < 0.05 were considered significant. All analyses were conducted using the R version 3.2.3 for Windows statistical software package.

## Results

### Baseline prognostic factors

**Fig. 1:**
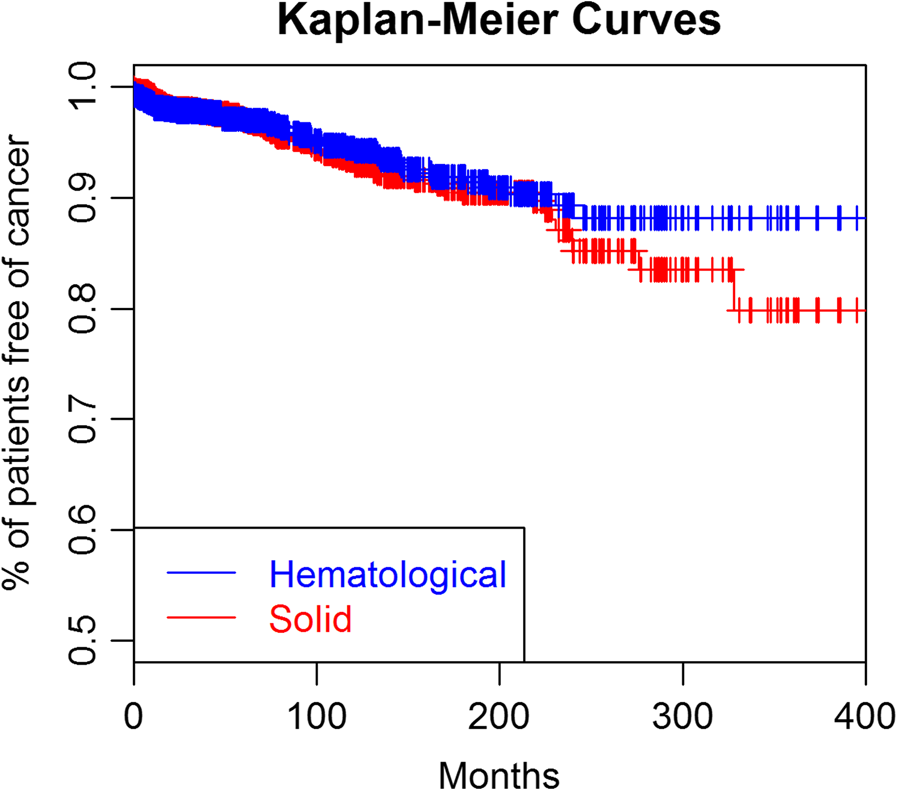
Survival curve for the development of solid and hematological cancer

Baseline characteristics of primary SjS patients are summarized in Table 1. The cohort consisted of 1300 patients, including 1201 (92.4%) women and 99 (7.6%) men (female:male ratio, 12:1), with a mean age at diagnosis of 55.1 (SD 15.4) years (range, 15.3–92.9 years). After a median follow-up of 66.1 months (range 1–560.3 months; 9922.3 person-years), 127 (9.8%) patients developed 133 cancers: 64 patients developed a solid cancer, 57 patients hematological cancer, 4 patients both solid and hematological cancers, and 2 patients two different types of solid neoplasia. The most frequent types of cancers included B-cell MALT lymphomas (*n* = 27, 20%), other B-cell lymphomas (*n* = 19, 14%), breast (*n* = 14, 11%), colorectal (*n* = 9, 7%), myeloid neoplasia/leukemia (*n* = 8, 6%), lung (*n* = 6, 5%), and stomach (*n* = 5, 4%) cancers (Additional file 1: Table S1). In comparison with patients who developed solid cancer, those who developed hematological cancer had a higher frequency at diagnosis of cryoglobulins (*p* = 0.002), low C3 levels (*p* = 0.018), high ESSDAI score (*p* = 0.001), and high DAS (*p* < 0.001) (Additional file 1: Table S1). The median time to the development of cancer was 59.9 months. Figure 1 shows the Kaplan-Meier curves for the development of the first solid or hematological cancer. Survival (free of cancer) at 5, 10, 20, and 30 years was 93.9, 87.9, 76.9, and 70.3%, respectively. The following baseline variables at diagnosis were associated with hematological cancer in the Cox regression analysis (Table 1): anemia (HR 2.07; *p* = 0.009), C3 levels <0.82 g/L (HR 2.07; *p* = 0.033), C4 levels <0.11 g/L (HR 2.23; *p* = 0.015), monoclonal gammopathy (HR 2.13; *p* = 0.028), and cryoglobulins (HR 4.37; *p* < 0.001). Multivariate analysis identified anemia and cryoglobulins as variables independently associated with hematological cancer. No variables were significantly associated with the development of solid cancer.

**Table 1:** Baseline SjS-related features and risk of development of solid and hematological cancer

Baseline features	Values at diagnosis (*n* = 1300)	Solid cancer (*n* = 70)	Hematological cancer (*n* = 61)
Age at diagnosis	54.8 ± 15.4	†	†
Gender (male)	99 (7.6)	†	†
Ethnicity (white)	1246 (95.8)	0.41 [0.10–1.71]	0.99 [0.14–7.29]
Dry mouth	1299 (99.9)	–	–
Dry eye	1234 (94.9)	0.48 [0.19–1.20]	1.37 [0.33–5.63]
Altered ocular tests	1013/1156 (87.6)	1.29 [0.47–3.58]	5.38 [0.74–39.03]
Altered parotid scintigraphy	835/992 (84.2)	0.87 [0.41–1.86]	1.34 [0.53–3.41]
Positive salivary gland biopsy	497/629 (79)	0.92 [0.35–2.44]	1.01 [0.42–2.45]
Anemia (Hb < 110 g/L)	216/1297 (16.7)	1.05 [0.59–1.86]	*2.07 [1.20–3.58]* **‡**
Leukopenia (<4000/mm^3^)	227/1297 (17.5)	1.49 [0.87–2.55]	0.96 [0.50–1.86]
Thrombocytopenia (<150,000/mm^3^)	87/1297 (6.7)	1.18 [0.54–2.57]	1.17 [0.50–2.72]
Neutropenia (<1500/mm^3^)	137/1296 (10.6)	1.41 [0.74–2.71]	1.66 [0.83–3.32]
Lymphopenia (<1000/mm^3^)	141/1295 (10.9)	1.80 [0.96–3.35]	1.43 [0.71–2.86]
Antinuclear antibodies+	1124/1295 (86.8)	0.95 [0.49–1.85]	1.05 [0.50–2.21]
Rheumatoid factor+	583/1245 (46.8)	1.13 [0.71–1.82]	1.08 [0.65–1.80]
Anti-Ro/SS-A+	981/1294 (75.8)	1.43 [0.81–2.53]	1.28 [0.70–2.36]
Anti-La/SS-B+	626/1289 (48.6)	0.99 [0.62–1.60]	1.17 [0.69–1.97]
Monoclonal gammopathy	114/1018 (11.2)	1.94 [0.99–3.80]	*2.13 [1.08–4.19]*
Cryoglobulins+	70/929 (7.5)	0.70 [0.25–1.94]	*4.37 [2.32–8.22]* **‡**
Low C3 levels (<0.82 g/L)	144/1233 (11.7)	0.59 [0.21–1.62]	*2.07 [1.06–4.03]*
Low C4 levels (<0.11 g/L)	163/1218 (13.4)	1.07 [0.51–2.24]	*2.23 [1.17–4.26]*

Values are represented as HRs [95% CIs]

Set in italics, statistically significant (*p* < 0.05) SS-related features associated with cancer in the univariate Cox proportional hazards regression analysis adjusted for age at diagnosis and gender

*SjS* Sjögren syndrome

†Adjusting variable

‡Statistically significant (*p* < 0.05) SS-related features associated with cancer in the multivariate Cox proportional hazards regression analysis adjusted for age at diagnosis and gender

### Systemic activity

Table 2 summarizes the relationship between organ-by-organ baseline ESSDAI activity at diagnosis and the risk of hematological and solid cancer. The mean ESSDAI score at diagnosis of the entire cohort was 6.00 (SD 6.6) and correlated with the risk of hematological cancer (HR 1.06; *p* < 0.001); the highest risk was found in patients with a high baseline DAS (HR 4.34; *p* < 0.001). Baseline activity in the constitutional (HR 2.44; *p* = 0.011), lymphadenopathy (HR 8.03; *p* < 0.001), glandular (HR 3.04; *p* < 0.001), and biological (HR 2.12; *p* = 0.011) domains was associated with a higher risk of hematological cancer. Multivariate analysis showed that the lymphadenopathy and glandular domains were independently associated with the risk of hematological cancer. A sensitivity analysis comparing no/low activity vs. moderate/high activity showed similar results (although with higher HRs), with the addition of the hematological domain, which was significantly associated with the development of hematological cancer (HR 2.72; *p* = 0.001) (Additional file 1: Table S3). Additional file 1: Table S4 summarizes the mean baseline ESSDAI scores of patients classified according to the type of cancer.

**Table 2:** Association between organ-by-organ baseline ESSDAI activity at diagnosis and the risk of development of solid and hematological cancer

Variables	Values at diagnosis (*n* = 1295)^a^	Solid cancer (*n* = 70)	Hematological cancer (*n* = 61)
Baseline DAS			
Low	707 (54.6)	REF	REF
Moderate	426 (32.9)	1.17 [0.71–1.92]	1.37 [0.73–2.58]
High	162 (12.5)	0.66 [0.28–1.56]	*4.34 [2.35–8.00]*
ESSDAI domains†			
Constitutional	115 (8.9)	1.13 [0.48–2.62]	*2.44 [1.23–4.87]*
Lymphadenopathy	89 (6.9)	0.39 [0.10–1.60]	*8.03 [4.62–13.94]* **‡**
Glandular	179 (13.8)	*0.35 [0.14–0.87]*	*3.04 [1.78–5.18]* **‡**
Articular	498 (38.5)	0.95 [0.58–1.56]	1.30 [0.78–2.18]
Cutaneous	131 (10.1)	1.40 [0.70–2.83]	1.10 [0.47–2.57]
Pulmonary	102 (7.9)	1.58 [0.71–3.48]	1.07 [0.38–2.99]
Renal	23 (1.8)	1.21 [0.17–8.80]	–
Muscular	15 (1.2)	1.57 [0.22–11.4]	–
Peripheral nervous system	54 (4.2)	0.59 [0.14–2.45]	–
Central nervous system	41 (3.2)	–	0.44 [0.06–3.22]
Hematological	447 (34.5)	1.31 [0.81–2.10]	1.36 [0.81–2.26]
Biological	681 (52.6)	1.32 [0.81–2.15]	*2.12 [1.19–3.76]*

Values are represented as the HRs (95% CIs)

Set in italics, statistically significant (*p* < 0.05) ESSDAI domains associated with cancer in the univariate Cox proportional hazards regression analysis adjusted for age at diagnosis and gender

*REF* reference level, *ESSDAI* EULAR-Sjögren Syndrome Disease Activity Index, *DAS* disease activity states

^a^In 5 patients, there was not enough information to calculate the ESSDAI at diagnosis

‡Statistically significant (*p* < 0.05) ESSDAI domains associated with cancer in the multivariate Cox proportional hazards regression analysis adjusted for age at diagnosis and gender

†Level of activity is recoded as no versus any type of activity (low/moderate/high) in the analysis

Additional file 1: Table S5 summarizes the potential influence of SJS-related therapies (pilocarpine, hydroxychloroquine, corticosteroids, immunosuppressive agents, intravenous immunoglobulin, and rituximab) in the development of cancer. In the univariate analysis, we found a higher risk of developing hematological cancer in patients receiving corticosteroids (HR 2.67; *p* < 0.001) or an immunosuppressive agent (HR 2.83; *p* < 0.001), although the multivariate analysis adjusted for the main prognostic factors identified (anemia, monoclonal gammopathy, cryoglobulins, low C3, low C4, and ESSDAI) showed only non-significant differences.

### Prognostic factors for hematological cancer subtypes

According to the WHO classification, we classified hematological cancers into three main groups: B-cell MALT lymphoma (*n* = 27), B-cell non-MALT lymphoma (*n* = 19), and non-B-cell (*n* = 15) cancers (Table 3). Baseline prognostic factors associated with B-cell MALT lymphomas included cryoglobulins (HR 6.32; *p* < 0.001) and low C3 levels (HR 3.25; *p* = 0.010). For B-cell non-MALT lymphomas, the prognostic factors included anemia (HR 2.58; *p* = 0.047), monoclonal gammopathy (HR 3.45; *p* = 0.024), cryoglobulins (HR 3.34; *p* = 0.028), and low C4 levels (HR 3.83; *p* = 0.014). For non-B-cell hematological cancers, prognostic factors included anemia (HR 4.02; *p* = 0.009), neutropenia (HR 5.86; *p* = 0.002), thrombocytopenia (HR 4.85; *p* = 0.004), and cryoglobulins (HR 4.36; *p* = 0.034).

**Table 3:** Association between organ-by-organ baseline ESSDAI activity at diagnosis and the risk of development of the three main subtypes of hematological cancer

Variables	B-cell MALT (*n* = 27)	B-cell non-MALT (*n* = 19)	Non-B-cell neoplasms (*n* = 15)
Dry eye	–	0.90 [0.12–6.83]	0.76 [0.10–5.94]
Altered ocular tests	–	1.33 [0.17–10.17]	–
Altered parotid scintigraphy	–	1.10 [0.25–4.86]	0.49 [0.13–1.87]
Positive salivary gland biopsy	1.84 [0.42–8.04]	0.37 [0.07–2.12]	0.83 [0.17–3.98]
Anemia (Hb < 110 g/L)	0.89 [0.31–2.61]	*2.58 [1.01–6.58]*	*4.02 [1.42–11.38]*
Leukopenia (<4000/mm^3^)	0.51 [0.15–1.69]	0.84 [0.24–2.89]	2.54 [0.86–7.52]
Thrombocytopenia (<150000/mm^3^)	0.47 [0.06–3.48]	–	*4.85 [1.64–14.34]*
Neutropenia (<1500/mm^3^)	1.24 [0.42–3.63]	0.51 [0.07–3.92]	*5.86 [1.89–18.14]*
Lymphopenia (<1000/mm^3^)	2.12 [0.79–5.68]	0.32 [0.04–2.48]	2.14 [0.65–7.02]
Antinuclear antibodies+	1.15 [0.35–3.84]	0.86 [0.25–2.97]	1.14 [0.26–5.10]
Rheumatoid factor+	0.94 [0.43–2.03]	1.16 [0.47–2.86]	1.28 [0.46–3.53]
Anti-Ro/SS-A+	1.07 [0.42–2.71]	1.63 [0.53–5.03]	1.26 [0.39–4.09]
Anti-La/SS-B+	0.66 [0.30–1.46]	2.35 [0.88–6.32]	1.34 [0.47–3.83]
Monoclonal gammopathy	1.30 [0.38–4.43]	*3.34 [1.14–9.83]*	2.16 [0.58–8.04]
Cryoglobulins+	*6.32 [2.47–16.15]*	*3.31 [1.06–10.36]*	*4.36 [1.12–16.99]*
Low C3 levels (<0.82 g/L)	*3.25 [1.33–7.93]*	1.14 [0.26–5.06]	1.43 [0.31–6.48]
Low C4 levels (<0.11 g/L)	1.76 [0.65–4.78]	*3.83 [1.31–11.21]*	1.36 [0.30–6.11]
Baseline ESSDAI	*1.07 [1.03–1.11]*	1.04 [0.98–1.09]	*1.08 [1.03–1.13]*
Baseline DAS (REF = low)			
Moderate	0.91 [0.33–2.51]	1.20 [0.42–3.47]	3.37 [0.84–13.52]
High	*4.80 [1.99–11.62]*	2.60 [0.84–8.07]	*8.29 [2.05–33.55]*
ESSDAI domains†			
Constitutional	1.31 [0.39–4.44]	1.62 [0.37–7.12]	*7.20 [2.43–21.32]*
Lymphadenopathy	*9.39 [4.20–21.02]*	*8.99 [3.35–24.14]*	*4.74 [1.32–17.02]*
Glandular	*7.35 [3.36–16.10]*	1.34 [0.44–4.13]	1.38 [0.38–5.04]
Articular	1.09 [0.50–2.39]	2.07 [0.82–5.18]	1.00 [0.34–3.00]
Cutaneous	1.16 [0.35–3.87]	1.28 [0.29–5.56]	0.78 [0.10–6.00]
Pulmonary	–	–	*4.98 [1.50–16.59]*
Central nervous System	1.12 [0.15–8.29]	–	–
Hematological	1.10 [0.50–2.42]	0.61 [0.22–1.69]	*4.99 [1.58–15.76]*
Biological	1.35 [0.60–3.06]	*6.57 [1.51–28.55]*	1.68 [0.57–4.95]

Values are represented as the HRs (95% CIs). B-cell non-MALT lymphomas included diffuse large B-cell lymphoma (7), marginal zone lymphoma (4), myeloma multiple (2), and other B-cell lymphomas (6). Non-B-cell neoplasms included myelodysplastic syndromes (4), leukemia (4), Hodgkin lymphoma (4), and T/NK cell neoplasias (3)

Set in italics, statistically significant (*p* < 0.05) SS-related features and ESSDAI domains associated with cancer in the univariate Cox proportional hazards regression analysis adjusted for age at diagnosis and gender

*REF* reference level, *ESSDAI* EULAR-Sjögren Syndrome Disease Activity Index, *DAS* disease activity states

†Level of activity is recoded as no versus any type of activity (low/moderate/high) in the analysis. There were not enough observations to fit models for the renal, muscular, and peripheral nervous system domains

Baseline systemic activity (global ESSDAI score) and high DAS were associated with a higher risk of B-cell MALT and non-B-cell cancers (but not B-cell non-MALT lymphomas). Distribution per organ showed that baseline systemic activity in the lymphadenopathy domain was linked to a higher risk for the three subtypes of hematological cancer, activity in the glandular domain with a higher risk of MALT lymphomas, activity in the biological domain with a higher risk of non-MALT B-cell lymphomas, and activity in the constitutional, pulmonary, and hematological domains with a higher risk of non-B-cell hematological cancer (Table 3).

### Standardized incidence ratios (SIRs) for cancer

For all cancers combined, the SIR estimate was 1.91 (95% CI 1.60 to 2.28) and was higher in men than in women (2.29 vs. 1.87) (Table 4).The SIR was 1.13 (95% CI 0.88 to 1.46) for solid cancer and 11.02 (95% CI 8.35 to 14.54) for hematological cancer. With respect to solid cancers, we found an increased risk for thyroid cancer (SIR 5.17; 95% CI 1.94 to 13.79), cancers of the lip and oral cavity (SIR 4.81; 95% CI 1.81 to 12.83), and stomach cancer (SIR 2.53; 95% CI 1.05 to 6.07) in women. We analyzed potential predictive factors for the development of thyroid, lip, and oral cavity, and stomach cancers (Additional file 1: Table S6) and found that only ethnicity was a predictive factor for the development of any of these cancers, since non-white patients had a HR of 10.44 (*p* = 0.004).

**Table 4:** Standardized incidence ratios (SIRs) for cancer classified according to the GLOBOCAN categories

Cancer categories	Total (*n* = 1239)^a^					Women (*n* = 1145)					Men (*n* = 94)				
	Obs‡	Exp	SIR	LCI	UCI	Obs	Exp	SIR	LCI	UCI	Obs	Exp	SIR	LCI	UCI
All cancers†	121	63.77	1.9	1.59	2.27	105	56.77	1.85	1.53	2.24	16	7	2.29	1.4	3.73
Solid cancers§	60	53.1	1.13	0.88	1.46	55	47.42	1.16	0.89	1.51	5	5.68	0.88	0.37	2.12
Thyroid	4	0.79	5.05	1.89	13.45	4	0.77	5.17	1.94	13.79	0	0.02	0	–	–
Lip, oral cavity	4	0.99	4.05	1.52	10.8	4	0.83	4.81	1.81	12.83	0	0.16	0	–	–
Stomach	5	2.24	2.23	0.93	5.36	5	1.98	2.53	1.05	6.07	0	0.26	0	–	–
Kidney	3	1.67	1.8	0.58	5.57	2	1.44	1.39	0.35	5.55	1	0.23	4.39	0.62	31.14
Brain, nervous system	2	1.17	1.72	0.43	6.86	2	1.07	1.87	0.47	7.48	0	0.1	0	–	–
Gallbladder	1	0.77	1.3	0.18	9.2	1	0.72	1.39	0.2	9.83	0	0.05	0	–	–
Lung	6	4.66	1.29	0.58	2.87	4	3.43	1.17	0.44	3.11	2	1.23	1.63	0.41	6.5
Prostate	2	1.66	1.2	0.3	4.81						2	1.66	1.2	0.3	4.81
Melanoma of skin	2	1.73	1.16	0.29	4.62	2	1.63	1.23	0.31	4.92	0	0.1	0	–	–
Bladder	2	2.1	0.95	0.24	3.8	2	1.48	1.35	0.34	5.42	0	0.63	0	–	–
Colorectal	9	10.08	0.89	0.46	1.72	9	9.02	1	0.52	1.92	0	1.06	0	–	–
Breast	14	15.68	0.89	0.53	1.51	14	15.68	0.89	0.53	1.51					
Pancreas	2	2.29	0.87	0.22	3.5	2	2.1	0.95	0.24	3.8	0	0.18	0	–	–
Cervix uteri	1	1.39	0.72	0.1	5.1	1	1.39	0.72	0.1	5.1					
Corpus uteri	2	3.72	0.54	0.13	2.15	2	3.72	0.54	0.13	2.15					
Ovary	1	2.16	0.46	0.07	3.28	1	2.16	0.46	0.07	3.28					
Hematological^b^	50	4.54	11.02	8.35	14.54	42	4.14	10.14	7.49	13.72	8	0.39	20.34	10.17	40.67
Leukemia	3	1.49	2.02	0.65	6.26	2	1.34	1.49	0.37	5.95	1	0.14	7.02	0.99	49.8
MM/ID and lymphoma	47	3.05	15.41	11.58	20.51	40	2.80	14.29	10.48	19.48	7	0.25	27.91	13.31	58.54
Hodgkin lymphoma	4	0.21	19.41	7.29	51.72	3	0.19	15.87	5.12	49.21	1	0.02	58.64	8.26	416.27
MM/ID	31	0.86	36.17	25.44	51.43	28	0.79	35.59	24.58	51.55	3	0.07	42.58	13.73	132.03
Non-Hodgkin lymphoma	12	1.99	6.04	3.43	10.64	9	1.82	4.94	2.57	9.49	3	0.16	18.37	5.93	56.96

*Obs* observed, *Exp* expected, *SIR* standardized incidence ratios, *LCL* lower confidence limit, *UCL* upper confidence limit, *MM/ID* myeloma multiple and malignant immunoproliferative diseases

^a^Excluding patients diagnosed with cancer before fulfillment of primary SjS criteria (*n* = 55) and patients with an age at SjS diagnosis <15 years (*n* = 6) (the age category “0–14” was excluded when SIR were computed). From 6 patients with an age at SjS diagnosis <15 years, one was diagnosed with cancer

^b^Excluding 7 patients with hematological neoplasia not included in the GLOBOCAN categories (1 refractory cytopenia with multilineage dysplasia, 1 refractory anemia with unilineage dysplasia, 1 refractory anemia with excess blasts, 1 myelodisplastic syndrome unclassifiable, 1 mastocytosis, 1 essential thrombocythemia, and 1 angioimmunoblastic T-cell lymphoma) (see Additional file 1: Table S5) and 4 patients who developed first a solid cancer and subsequently a hematological cancer

†Excluding patients with non-melanoma skin cancer (*n* = 4, not included in the GLOBOCAN classification) and a non-classifiable skin cancer (*n* = 1, uncertain whether was a melanoma or non-melanoma cancer)

§Excluding 4 patients with neoplasia not included in the GLOBOCAN categories (2 endocrine, 2 ocular)

With respect to hematological cancers, we had to adapt our classification of hematological cancer (based on the WHO 2016 nomenclature) to the four categories of hematological cancer used in the GLOBOCAN database (which are based on ICD codes); the correspondence between the two classifications is shown in Additional file 1: Table S7. We found an increased risk for three of the four GLOBOCAN categories corresponding to hematological cancer: multiple myeloma and immunoproliferative diseases (SIR 36.17; 95% CI 25.44 to 51.43), Hodgkin lymphoma (SIR 19.41; 95% CI 7.29 to 51.72), and non-Hodgkin lymphoma (SIR 6.04; 95% CI 3.43 to 10.64).

## Discussion

In non-specialized medical settings, primary SjS is often considered a chronic, non-life threatening disease that causes dryness, fatigue, and pain. However, systemic involvement has increasingly been recognized as a key part of the disease spectrum with a significant weight in dictating the prognosis and survival [13]. Among the systemic manifestations of SjS, lymphoma is one of the worst complications that physicians should expect. In 1978, Kassan et al. [2] estimated a SIR for NHL of 44.4 in patients with primary SjS. The SIRs estimated by subsequent studies, even though the great majority have been lower, have confirmed that primary SjS patients are at higher risk of lymphoma, with a pooled 14-fold higher risk reported by Liang et al. [19] in a recent meta-analysis. However, this meta-analysis included cohorts of patients in whom the diagnosis of primary SjS was based on the 1993 criteria, whose fulfillment allows the inclusion of patients with negative Ro/La antibodies and negative biopsy. This creates a significant bias, since several studies have reported a lower risk of lymphoproliferation in immunonegative patients [8, 20]. Table 5 summarizes the studies based on the fulfillment of the 2002 criteria [8–11, 21–27]. The SIRs for B-cell lymphoma range between 7 and 9 in population-based studies and between 16 and 48 in hospital-based studies. The majority of these studies were focused only on B-cell lymphoma, and most of them used an old terminology (NHL).

**Table 5:** Studies analyzing cancer risk in patients with primary SjS based on the fulfillment of the 2002 criteria

Author	Year	Country	Setting	Primary SjS patients (*n*)	Mean folow-up (yrs/pat-years)	Population cancer registry/classif	Hemat neoplasia classif	Cancer (*n*)	All-cancer SIR (95% CI)	Specific solid cancer SIR	Hemat cancers (*n*)	NHL (*n*)	MALT (*n*)	DLBC (*n*)	MZ (*n*)	Myel (*n*)	Other B-cell	Non-B-cell	NHL SIR (95% CI)	MM SIR (95% CI)
Theander	2006	Sweden	Hosp	286	jul-64	Swedish Cancer Reg/ICD7	WHO 2001	33	1.42 (0.98–2.00)	NA	12	11	1	7	0	2	1	T-cell (1)	15.6 (7.8–27.8)	3.27 (0.1–18.2)
Baimpa	2009	Greece	Hosp	536	2.6/ND	NA	ND	ND	NA	NA	40	38	21	7	5	0	5	HD (1), T-cell (1)	NA	ND
Zhang	2010	China	Hosp	1320	4.4/ND	Shangai Reg/ICD10	WHO 2001	29	3.25 (2.12–4.52)	NA	10	8	2	2	0	0	3	T-cell (1)	48.1 (20.7–94.8)	37.9 (4.58–136.7)
Weng	2012	Taiwan	Pop	7852	ND/27246	NHI/ICD9	ICD9	227	1.01 (0.74–1.35)	Colon 0.22 (0.05–0.6); Thyroid 2.56 (1.4–4.3)	31	23	ND	ND	ND	ND	ND	ND	7.1 (4.2–10.3)	6.1 (2.0–14.2)
Baldini	2012	Italy	Hosp	563	6/ND	NA	WHO 2001	NA	NA	NA	NA	12	8	3	1	0	0	ND	NA	ND
Hemminki	2012	Sweden	Pop	1516	ND/16700	Swedish Cancer Reg/ICD	NA	NA	NA	Breast 0.46 (0.26–0.75)	NA	NA	ND	ND	ND	ND	ND	ND	NA	ND
Johnsen	2013	Norway	Pop	443	ND/3813	Norway Reg/ICD10	ICD10	NA	NA	NA	NA	7	6	0	1	0	0	ND	9.0 (7.1–26.3)	ND
Risselada	2013	Netherl	Hosp	195	7.7/ND	NA	WHO 2001	NA	NA	NA	NA	21	10	8	3	0	0	ND	NA	ND
Quartuccio	2014	Italy	Hosp	661	ND	NA	ND	NA	NA	NA	NA	40	ND	ND	ND	ND	ND	ND	NA	ND
Papageorgiou	2015	Greece	Hosp	ND	ND	ND	WHO	ND	ND	ND	ND	77	51	12	8	0	6		ND	
Nocturne	2016	France	Hosp	ND	ND	ND	WHO	ND	ND	ND	ND	99	58	17	18	0	6	HD (1), T-cell (1)	ND	ND
Present study	2016	Spain	Hosp	1300	7.6/ND	GLOBOCAN/ICD10	WHO 2016	122	1.91 (1.6–2.28)	Thyroid 5.17 (1.94–13.79); Lip/oral 4.81 (1.81–12.83); Stomach 2.53 (1.05–6.07)	61	12	27	7	4	2	6	HD (4), T-cell (3), myeloid/leuk (8)	6.04 (3.43–10.64)	36.17 (25.44–51.43)

*ND* not detailed, *NA* non-applicable, *MALT* mucosa-associated lymphoid tissue, *DLBC* Diffuse large B-cell lymphoma, *MZ* marginal zone lymphoma, *MM* myeloma multiple, *Netherl* Netherlands, *Hosp* hospital, *Pop* population, *Classif* classification, *Hemat* hematological, *Myel* Myeloma

The present study is the first to analyze the risk of the different types of cancer (including solid cancer and hematological cancers other than lymphoma) and identify the corresponding baseline predictive factors in a hospital-based cohort of patients with primary SjS. Only three previous studies have estimated the SIR for all-type of cancers in primary SjS. The risk was not significant in a population-based study [11] but was significant in the two hospital-based studies [8, 21] (although the significance was at the limit in the study by Theander et al [8]). We also found a higher significant risk with a SIR of nearly 2, although when we separated solid and hematological cancers, the SIR for solid cancers was not significant, while the SIR for hematological cancer was 11-fold higher (10-fold higher in women and 20-fold higher in men).

Although the vast majority of cells infiltrating the salivary glands of patients with primary SjS are T cells [6], the majority of lymphomas reported are of B-cell origin (in our study, the ratio between B- and T-cell lymphomas was 15:1). Among B-cell lymphomas, the different subtypes not only have differing clinical presentations but also have a different frequency and, logically, a different prognosis. Therefore, the identification of predictive factors for the development of the different subtypes of B-cell lymphoma seems rational. Three subtypes of lymphoma alone account for more than 90% of reported cases in primary SjS (Table 5): MALT lymphoma (58%), DLBC (20%), and MZ lymphoma (13%). Plasma-cell myeloma was reported in only four cases (2%), although a recent study [28] has described an increased risk in primary SjS patients presenting with monoclonal gammopathy. Among non-B-cell hematological cancers, we found 8 patients with myeloid neoplasia/leukemia (not reported in previous studies), four cases of Hodgkin disease, and three cases of T/NK-cell lymphoma (4 cases and 2 cases previously reported, respectively) [8–10, 21], representing 25% of the hematological cancers observed.

Recent studies have confirmed severe parotid involvement, purpura, leukopenia, anti-La antibodies, raised levels of BAFF and beta2-microglobulin, cryoglobulins, monoclonal band, and hypocomplementemia as risk factors for B-cell lymphoma [24, 25, 29, 30]. The present study is the first to report that the prognostic role of these risk factors may differ according to the subtype of hematological cancer. We found a higher risk of MALT lymphoma in patients presenting with systemic activity, positive cryoglobulins, and low C3 levels at SjS diagnosis, while the risk of non-MALT B-cell lymphomas was unrelated to systemic activity, with anemia, monoclonal gammopathy, cryoglobulins, and low C4 levels at SjS diagnosis being the main risk factors. For non-B-cell hematological cancers, risk factors were systemic activity, cytopenias (anemia, thrombocytopenia and leukopenia), and cryoglobulins at SjS diagnosis. With respect to the influence of immunosuppressive therapies in the development of cancer, we found a higher risk of developing hematological cancer in patients who ever received corticosteroids/immunosuppressants, although the differences were non-significant after adjustment for the main predictive factors in which systemic activity is included. The explanation for the disappearance of significant differences in the multivariate model is that the more active patients are those who are mainly treated with corticosteroids/immunosuppressants and, therefore, the adjustment of comparisons for systemic activity yielded non-significant differences.

Analysis of systemic organ-by-organ activity showed that activity in the lymphadenopathy domain at SjS diagnosis was associated with a high risk of all subtypes of hematological neoplasia, while activity in the glandular domain was associated with the development of MALT lymphoma, activity in the biological domain with non-MALT lymphoma, and activity in the constitutional, pulmonary, and hematological domains with non-B-cell cancers. These differentiated high-risk profiles (Additional file 2: Figure S1), which are present at the diagnosis of primary SjS, may help physicians identify which patients may be at high risk of developing a specific subtype of hematological cancer. Together with the differentiated clinical presentation and the predilection for specific lymphoma subtypes, these high-risk profiles may contribute to an as-early-as-possible diagnosis and, therefore, to earlier specific therapeutic management that could improve survival.

We also analyzed and characterized the development of solid neoplasia in our cohort of patients with primary SjS and found that the risk was not increased with respect to the control population, in contrast to the results reported in Chinese patients [21]. Only two previous studies analyzed the risk for specific types of solid cancer and reported a lower risk for the development of colon and breast cancers [11, 27] and a higher risk for thyroid cancer [11]. We found an enhanced risk for the development of thyroid, lip/oral cavity, and stomach cancers (SIRs of 5.17, 4.81, and 2.53, respectively), with an enhanced risk of developing any of these three cancers (HR 10.44) found in non-white patients. The highest risk was for thyroid cancer, in which several risk factors [31] are clearly shared with primary SjS, including the predominantly female involvement [32], the low frequency of smokers [33], and the high frequency of association with autoimmune thyroiditis [34, 35]. The finding of the higher risk for cancers of the oral cavity and stomach in women with primary SjS is interesting, since the oral cavity is overwhelmingly involved in primary SjS and the stomach is the most frequent extraglandular extranodal site of lymphoma involvement in primary SjS [36], although further specific studies of the potential influence of dietary and other lifestyle factors (not analyzed in our study) are necessary. We also explored a potential association between the degree of oral involvement and the risk of developing cancer of the lip/oral cavity, but found no significant association between dry mouth, the severity of parotid scintigraphy results, and the presence of lymphocytic sialoadenitis in the salivary biopsy (data not shown), although the number of patients was too small to make solid conclusions.

Several methodological considerations should be discussed with respect to our results. The first is the limitation of the database available to estimate the SIRs for hematological cancer in the Spanish population (GLOBOCAN), since the two main GLOBOCAN categories (NHL and MM/ID) contain a mix of B-cell subtypes. In the case of primary SjS, the inclusion of the most frequent hematological cancer (MALT) in the MM/ID category and not in the NHL category may cause some misinterpretation of the corresponding SIRs (31 for MM/ID, 12 for NHL); in fact, the SIR for the combination of the three categories of lymphomas included in GLOBOCAN was 15.41 (95% CI 11.58–20.51). In addition, 11 of the 61 hematological cancers developed by our primary SjS patients were not included in the GLOBOCAN categories corresponding to the hematological cancers, leading to a probable underestimation of the global SIR for hematological neoplasia found in our study. This highlights the importance of detailed information in these types of studies on the correspondence between the classification of hematological neoplasia often used in clinical practice (WHO) and the classification used in the cancer database of the general population (overwhelmingly using ICD codes). In addition, a potential risk of selection bias between the different types of hematological cancers should be taken into account (tertiary care centers will probably report a wider spectrum of cancer subtypes than less specialized centers). Homogeneity in classifying SjS patients according to the most accepted criteria (2002 AECG) is important, a factor not applied in recent studies that used the older 1993 criteria [37, 38] or in which the diagnosis of SjS was self-reported by the patient [39–41].

## Conclusions

Patients with primary SjS had an 11-fold higher risk of developing hematological cancers than the general Spanish population. One third of the cancers developed during the study follow-up period were B-cell lymphomas (of which MALT lymphomas accounted for 60% of cases); the main prognostic factors identified at SjS diagnosis included systemic activity, cytopenias, and cryoglobulin-related immunological markers, although the weight of these factors differed for each subtype of B-cell lymphoma. Primary SjS was also associated with a higher risk of some types of non-hematological cancers (thyroid, oral cavity, and stomach). Patients with primary SjS should be closely followed not only for the enhanced risk of hematological cancer (not only B-cell lymphomas), but also for some types of solid cancer.

## Additional file

Additional file 1:
**Table S1.** Frequency of solid and hematologic cancers. **Table S2.** Comparison between patients who developed solid cancer and those who developed hematological cancer. **Table S3.** Sensitivity analysis comparing no/low activity vs. moderate/high activity. **Table S4.** Mean baseline ESSDAI scores of patients classified according to the type of cancer. **Table S5.** Analysis of a potential influence of SJS-related therapies (pilocarpine, hydroxychloroquine, corticosteroids, immunosuppressive agents, intravenous immunoglobulin and rituximab) in the development of cancer. **Table S6.** Analysis of potential predictive factors for the development of these three non-hematological cancers (thyroid, lip and oral cavity, and stomach cancers). **Table S7.** Correlation between the WHO 2016 nomenclature and the four categories of hematological cancer used in the GLOBOCAN database (which are based on ICD codes). (DOCX 38 kb)
Additional file 2: Figure S1. Frequency of the main WHO subtypes of hematological cancer and the corresponding predictive factors identified at SjS diagnosis. (TIF 182 kb)

## Abbreviations

DAS: Disease activity states
MALT: Mucosa-associated lymphoid tissue
SIR: Standardized incidence ratios
SjS: Sjögren syndrome
