# Supplementary material for: Characterization and risk estimate of cancer in patients with primary Sjögren syndrome
Source: J Hematol Oncol. 2017 Apr 17;10:90. doi: 10.1186/s13045-017-0464-5 (PMC5392920; doi:10.1186/s13045-017-0464-5)
Supplement: Additional file 1: — Table S1. Frequency of solid and hematologic cancers. Table S2. Comparison between patients who developed solid cancer and those who developed hematological cancer. Table S3. Sensitivity analysis comparing no/low activity vs. moderate/high activity. Table S4. Mean baseline ESSDAI scores of patients classified according to the type of cancer. Table S5. Analysis of a potential influence of SJS-related therapies (pilocarpine, hydroxychloroquine, corticosteroids, immunosuppressive agents, intravenous immunoglobulin and rituximab) in the development of cancer. Table S6. Analysis of potential predictive factors for the development of these three non-hematological cancers (thyroid, lip and oral cavity, and stomach cancers). Table S7. Correlation between the WHO 2016 nomenclature and the four categories of hematological cancer used in the GLOBOCAN database (which are based on ICD codes). (DOCX 38 kb) [file 13045_2017_464_MOESM1_ESM.docx]

**Additional file 1**

**Table S1.**

| **Types of cancer** | **n (%)** |
| --- | --- |
| Solid cancers (GLOBOCAN categories)* | N = 70 |
| Breast | 14 (20.3) |
| Colorectum | 9 (13.0) |
| No category | 9 (13.0) |
| Lung | 6 (8.7) |
| Stomach | 5 (7.2) |
| Lip, oral cavity | 4 (5.8) |
| Thyroid | 4 (5.8) |
| Prostate | 4 (5.8) |
| Kidney | 3 (4.3) |
| Bladder | 2 (2.9) |
| Brain, nervous system | 2 (2.9) |
| Melanoma of skin | 2 (2.9) |
| Pancreas | 2 (2.9) |
| Corpus uteri | 1 (1.4) |
| Cervix uteri | 1 (1.4) |
| Gallbladder | 1 (1.4) |
| Ovary | 1 (1.4) |
| Hematological cancers (WHO categories) | N = 61 |
| B cell | 46 (75.4) |
| MALT | 27 (44.3) |
| Other B-cell | 19 (31.1) |
| Myeloid/leukemias | 8 (13.1) |
| Hodgkin | 4 (6.6) |
| T/NK cell | 3 (4.9) |

**Table S2***

| **Variable** | **Solid cancer (n=66)** | **Hematological cancer (n=57)** | **P-value** |
| --- | --- | --- | --- |
| Mean age (years) | 59.4 ± 13.3 | 56.3 ± 14.2 | 0.216 |
| Gender (male) | 5 (7.6) | 9 (15.8) | 0.168 |
| Ethnicity (white) | 64 (97) | 56 (98.2) | 1.000 |
| Dry mouth | 66 (100) | 57 (100) | 1.000 |
| Dry eye | 61 (92.4) | 55 (96.5) | 0.449 |
| Altered ocular tests | 53/57 (93) | 49/50 (98) | 0.369 |
| Altered parotid scintigraphy | 42/50 (84) | 35/40 (87.5) | 0.767 |
| Positive salivary gland biopsy | 23/28 (82.1) | 28/34 (82.4) | 1.000 |
| Anaemia (Hb < 110 g/L) | 13 (19.7) | 18 (31.6) | 0.149 |
| Leukopenia (<4000/mm^3^) | 17 (25.8) | 10 (17.5) | 0.383 |
| Thrombocytopenia (<150000/ mm^3^) | 6 (9.1) | 5 (8.8) | 1.000 |
| Neutropenia (<1500/mm^3^) | 10 (15.2) | 9 (15.8) | 1.000 |
| Lymphopenia (<1000/mm^3^) | 9 (13.6) | 7 (12.3) | 1.000 |
| Antinuclear antibodies± | 56 (84.8) | 49 (86) | 1.000 |
| Rheumatoid factor± | 35/65 (53.8) | 31 (54.4) | 1.000 |
| Anti-Ro/SS-A± | 49/65 (75.4) | 41/55 (74.5) | 1.000 |
| Anti-La/SS-B± | 30/65 (46.2) | 27/55 (49.1) | 0.855 |
| Monoclonal gammopathy | 11/48 (22.9) | 11/45 (24.4) | 1.000 |
| Cryoglobulins± | 3/55 (5.5) | 13/43 (30.2) | **0.002** |
| Low C3 levels (<0.82 g/L) | 3/64 (4.7) | 10/52 (19.2) | **0.018** |
| Low C4 levels (<0.11 g/L) | 7/64 (10.9) | 11/51 (21.6) | 0.131 |
| Baseline ESSDAI | 5.7 ± 5.8 | 10.1 ± 7.5 | **0.001** |
| Baseline DAS |  |  | **<0.001** |
| Low | 36 (54.5) | 21 (36.8) |  |
| Moderate | 24 (36.4) | 15 (26.4) |  |
| High | 6 (9.1) | 21 (36.8) |  |
| * 4 patients who developed two types of cancers (hematological and solid) during follow up were excluded. | | | |

**Table S3.**

| **ESSDAI domains†** | **Values at diagnosis (n=1295)*** | **Solid cancer (n=70)** | **Hematological cancer (n=61)** |
| --- | --- | --- | --- |
|  |  |  |  |
| Constitutional | 24/1295 (1.9) | - | **7.30 [2.89-18.41]** |
| Lymphadenopathy | 36/1295 (2.8) | 0.86 [0.21-3.53] | **12.38 [6.79-22.57]** |
| Glandular | 37/1295 (2.9) | 0.41 [0.06-2.98] | **7.85 [4.01-15.36]** |
| Articular | 100/1295 (7.7) | 0.48 [0.15-1.53] | 1.78 [0.87-3.65] |
| Cutaneous | 116/1295 (9) | 1.34 [0.64-2.81] | 0.98 [0.39-2.45] |
| Pulmonary | 54/1295 (4.2) | 1.86 [0.67-5.20] | 1.01 [0.24-4.19] |
| Renal | 12/1295 (0.9) | 2.02 [0.28-14.67] | - |
| Muscular | 11/1295 (0.8) | - | - |
| Peripheral nervous system | 38/1295 (2.9) | 0.44 [0.06-3.21] | - |
| Central nervous system | 41/1295 (3.2) | - | 0.44 [0.06-3.22] |
| Haematological | 104/1295 (8) | 1.48 [0.77-2.84] | **2.72 [1.47-5.03]** |
| Biological | 257/1295 (19.8) | 1.07 [0.61-1.88] | **1.96 [1.15-3.34]** |
| Values are represented as HRs (95% CIs). | | | |
| In bold, statistically significant (p<0.05) ESSDAI domains associated with cancer in the univariate Cox proportional hazards regression analysis adjusted for age at diagnosis and gender. | | | |
|  |  |  |  |
| * In 5 patients there was not enough information to calculate the ESSDAI at diagnosis. | | | |
| † Level of activity is recoded as no/low activity versus moderate/high in the analysis. | | | |
| ESSDAI, EULAR- Sjögren Syndrome Disease Activity Index. | | | |

**Table S4**

| **System specific cancer** | **Baseline ESSDAI** | **Baseline DAS** | | |
| --- | --- | --- | --- | --- |
|  |  | **Low** | **Moderate** | **High** |
| Lymphoma | 10.0 ± 7.5 | 18 (36) | 14 (28) | 18 (36) |
| Skin | 10.0 ± 8.8 | 2 (28.6) | 3 (42.9) | 2 (28.6) |
| Leukemia | 8.8 ± 9.0 | 1 (25) | 2 (50) | 1 (25) |
| Myeloproliferative | 8.5 ± 6.6 | 2 (50) | 1 (25) | 1 (25) |
| Pulmonary | 7.8 ± 9.1 | 3 (50) | 2 (33.3) | 1 (16.7) |
| Gynecological | 7.1 ± 6.4 | 8 (44.4) | 6 (33.3) | 4 (22.2) |
| Neurological | 7.0 ± 1.4 | 0 (0) | 2 (100) | 0 (0) |
| Oropharynx | 5.8 ± 4.3 | 1 (25) | 3 (75) | 0 (0) |
| No cancer | 5.7 ± 6.5 | 628 (56.4) | 364 (32.7) | 121 (10.9) |
| Endocrine | 4.7 ± 4.2 | 5 (71.4) | 2 (28.6) | 0 (0) |
| Nephrourological | 4.3 ± 4.2 | 5 (71.4) | 2 (28.6) | 0 (0) |
| Ocular | 4.0 ± 1.4 | 1 (50) | 1 (50) | 0 (0) |
| Gastrointestinal | 3.6 ± 4.1 | 12 (75) | 4 (25) | 0 (0) |
| ESSDAI, EULAR- Sjögren Syndrome Disease Activity Index. | | | | |
| DAS, disease activity states. | | | | |

**Table S5**

|  |  | **Adjusted univariate analysis** | | **Adjusted multivariate analysis**** |
| --- | --- | --- | --- | --- |
| **Treatment** | **Patients ever treated (n=1300)** | **Solid cancer (n=70)** | **Hematological cancer (n=61)** | **Hematological cancer (n=61)** |
| Pilocarpine | 268 (20.6) | 0.63 [0.33-1.17] | 0.62 [0.31-1.23] | - |
| Hydroxychloroquine | 378 (29.1) | 0.80 [0.46-1.38] | 0.66 [0.35-1.22] | - |
| Corticosteroids | 522 (40.2) | 0.81 [0.50-1.31] | **2.67 [1.55-4.63]** | 1.41 [0.61-3.28] |
| Immunosuppressive agents | 217 (16.7) | 0.64 [0.32-1.29] | **2.83 [1.67-4.79]** | 1.25 [0.53-3.00] |
| Intravenous immunoglobulins | 33 (2.5) | 0.89 [0.22-3.64] | 0.42 [0.06-3.06] | - |
| Rituximab* | 19 (1.5) | 1.04 [0.14-7.52] | - | - |
| Values are represented as the HRs (95% CIs). | |  |  |  |
| In bold, statistically significant (p<0.05) treatments associated with cancer in the univariate Cox proportional hazards regression analysis adjusted for age at diagnosis and gender. | | | | |
| * 24 patients with lymphoma that requiered Rituximab therapy were excluded. | | | | |
| ** Multivariate Cox proportional hazards regression analysis adjusted for the age at diagnosis, gender and the main predictive factors for development of hematological neoplasia (anemia, monoclonal gammopathy, cryoglobulins, low C3, low C4 and ESSDAI domains). | | | | |

**Table S6**

| **Baseline features** | **Cancer (n=13)*** |
| --- | --- |
| Ethnia (non-white) | 10.44 [2.15-50.81] |
| Dry mouth | - |
| Dry eye | - |
| Altered ocular tests | - |
| Altered parotid scintigraphy | 1.38 [0.17-11.08] |
| Positive salivary gland biopsy | - |
| Anaemia (Hb < 110 g/L) | 0.76 [0.17-3.50] |
| Leukopenia (<4000/mm^3^) | 0.76 [0.17-3.46] |
| Thrombocytopenia (<150000/ mm^3^) | - |
| Neutropenia (<1500/mm^3^) | 0.59 [0.08-4.55] |
| Lymphopenia (<1000/mm^3^) | 0.79 [0.10-6.10] |
| Antinuclear antibodies+ | 0.83 [0.18-3.73] |
| Rheumatoid factor+ | 0.88 [0.29-2.63] |
| Anti-Ro/SS-A+ | 2.17 [0.47-10.03] |
| Anti-La/SS-B+ | 1.68 [0.54-5.16] |
| Monoclonal gammopathy | 1.81 [0.38-8.54] |
| Cryoglobulins+ | - |
| Low C3 levels (<0.82 g/L) | 0.88 [0.11-6.98] |
| Low C4 levels (<0.11 g/L) | 0.69 [0.09-5.46] |
| Baseline ESSDAI | 0.97 [0.88-1.07] |
| Baseline DAS | 0.97 [0.88-1.07] |
| ESSDAI domains† |  |
| Constitutional | 3.34 [0.91-12.27] |
| Lymphadenopathy | - |
| Glandular | 1.51 [0.41-5.59] |
| Articular | 0.28 [0.06-1.25] |
| Cutaneous | 1.61 [0.36-7.26] |
| Pulmonary | - |
| Renal | - |
| Muscular | - |
| Peripheral nervous system | - |
| Central nervous system | - |
| Haematological | 1.44 [0.48-4.30] |
| Biological | 0.93 [0.31-2.79] |
| Values are represented as HRs [95% CIs]. | |
| * Thyroid, lip and oral cavity and stomach cancers | |
| † Level of activity is recoded as no versus any type of activity (low/moderate/high) in the analysis. | |

**Table S7**

| **GLOBOCAN category** | **ICD-10 Code** | **ICD-10 Title** | **WHO definition** | **WHO classification** |
| --- | --- | --- | --- | --- |
| Hodgkin lymphoma | C81 | Hodgkin lymphoma | Hodgkin lymphoma | Hodgkin |
| Non-Hodgkin lymphoma | C82 | Follicular lymphoma | Follicular lymphoma | B cell |
| Non-Hodgkin lymphoma | C83 | Non-follicular lymphoma | Diffuse large B cell lymphoma, NOS | B cell |
| Non-Hodgkin lymphoma | C83 | Non-follicular lymphoma | Lymphoplasmacytic lymphoma | B cell |
| Non-Hodgkin lymphoma | C83 | Non-follicular lymphoma | Mantle cell lymphoma | B cell |
| Non-Hodgkin lymphoma | C84 | Lymphoid leukemia | Mature NK/T cell lymphoma | T/NK cell |
| Non-Hodgkin lymphoma | C84 | Mature T/NK-cell lymphomas | Peripheral T-cell lymphoma, NOS | T/NK cell |
| Non-Hodgkin lymphoma | C85 | Other specified and unspecified types of non-Hodgkin lymphoma | Primary mediastinal large B cell lymphoma | B cell |
| Multiple myeloma and malignant immunoproliferative diseases | C88 | Malignant immunoproliferative diseases and certain other B-cell lymphomas | MALT lymphoma | B cell |
| Multiple myeloma and malignant immunoproliferative diseases | C88 | Malignant immunoproliferative diseases and certain other B-cell lymphomas | Nodal marginal zone lymphoma | B cell |
| Multiple myeloma and malignant immunoproliferative diseases | C88 | Malignant immunoproliferative diseases and certain other B-cell lymphomas | Splenic marginal zone lymphoma | B cell |
| Multiple myeloma and malignant immunoproliferative diseases | C88 | Malignant immunoproliferative diseases and certain other B-cell lymphomas | Waldestrom macroglobulinemia | B cell |
| Multiple myeloma and malignant immunoproliferative diseases | C90 | Multiple myeloma and malignant plasma cell neoplasms | Plasma cell myeloma | B cell |
| Leukemias | C91 | Lymphoid leukemia | Chronic lymphocytic leukemia | B cell |
| Leukemias | C91 | Lymphoid leukemia | T-cell large granular lymphocytic leukemia | T/NK cell |
| Leukemias | C92 | Myeloid leukemia | Acute myelomonocytic leukemia | Myeloid/leukemias |
| Not included | C86 | Other specified types of T/NK-cell lymphoma | Angioimmunoblastic T-cell lymphoma | T/NK cell |
| Not included | D46 | Myelodysplastic syndromes | Myelodisplastic syndrome, unclassifiable | Myeloid/leukemias |
| Not included | D46 | Myelodysplastic syndromes | Refractory anemia with excess blasts | Myeloid/leukemias |
| Not included | D46 | Myelodysplastic syndromes | Refractory anemia with unlineage dysplasia | Myeloid/leukemias |
| Not included | D46 | Myelodysplastic syndromes | Refractory cytopenia with multilineage dysplasia | Myeloid/leukemias |
| Not included | D47 | Other leukemias of specified cell type | Essential thrombocythemia | Myeloid/leukemias |
| Not included | Q82 | Other leukemias of specified cell type | Mastocytosis | Myeloid/leukemias |
