# Supplementary figures and images for: Characterization and risk estimate of cancer in patients with primary Sjögren syndrome
Source: J Hematol Oncol. 2017 Apr 17;10:90. doi: 10.1186/s13045-017-0464-5 (PMC5392920; doi:10.1186/s13045-017-0464-5)

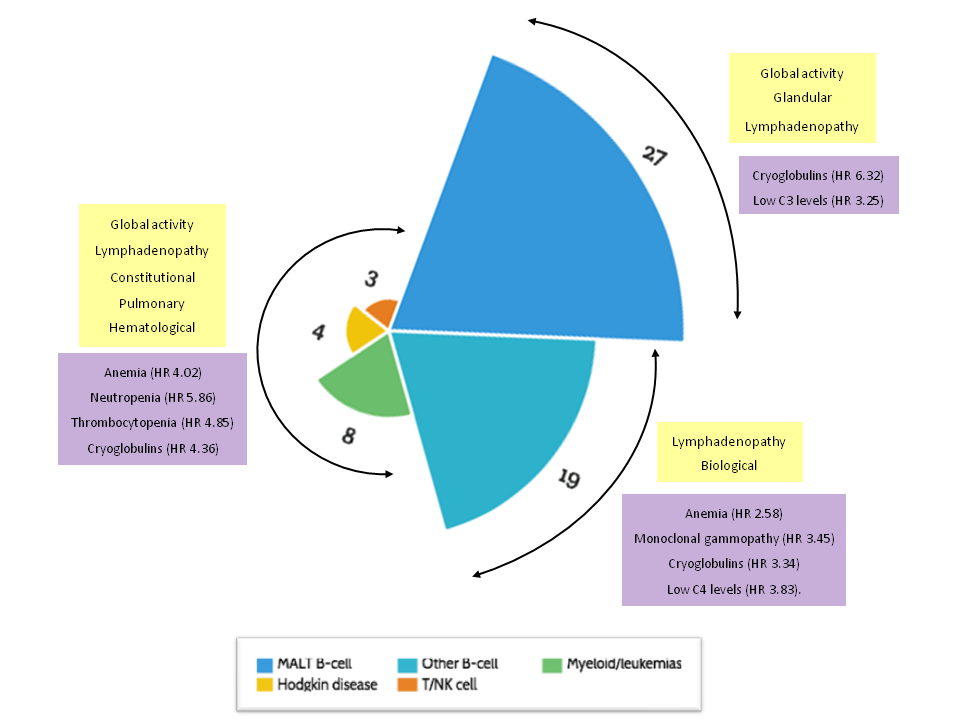

Supplement: Additional file 2: Figure S1. — Frequency of the main WHO subtypes of hematological cancer and the corresponding predictive factors identified at SjS diagnosis. (TIF 182 kb) [file 13045_2017_464_MOESM2_ESM.tif]
